# Improved Anthropomorphic Robotic Hand for Architecture and Construction: Integrating Prestressed Mechanisms with Self-Healing Elastomers

**DOI:** 10.3390/biomimetics10050284

**Published:** 2025-05-01

**Authors:** Mijin Kim, Rubaya Yaesmin, Hyungtak Seo, Hwang Yi

**Affiliations:** 1 Architectural Research of Technology & Scientific Design (ARTS) Lab, Seoul 02841, Republic of Korea; mijin07@korea.ac.kr; 2 Department of Architecture, College of Engineering, Korea University, Seoul 02841, Republic of Korea; 3 Department of Energy Systems Research, Ajou University, Suwon 16499, Republic of Korea; ry19@ajou.ac.kr; 4 Engineering Research Institute, Ajou University, Suwon 16499, Republic of Korea; 5 Department of Materials Science and Engineering, Ajou University, Suwon 16499, Republic of Korea

**Keywords:** robotic architecture, building construction, self-healing materials, soft robot, robot-arm gripper

## Abstract

Soft pneumatic robot-arm end-effectors can facilitate adaptive architectural fabrication and building construction. However, conventional pneumatic grippers often suffer from air leakage and tear, particularly under prolonged grasping and inflation-induced stress. To address these challenges, this study suggests an enhanced anthropomorphic gripper by integrating a pre-stressed reversible mechanism (PSRM) and a novel self-healing material (SHM) polyborosiloxane–Ecoflex™ hybrid polymer (PEHP) developed by the authors. The results demonstrate that PSRM finger grippers can hold various objects without external pressure input (12 mm displacement under a 1.2 N applied), and the SHM assists with recovery of mechanical properties upon external damage. The proposed robotic hand was evaluated through real-world construction tasks, including wall painting, floor plastering, and block stacking, showcasing its durability and functional performance. These findings contribute to promoting the cost-effective deployment of soft robotic hands in robotic construction.

## 1. Introduction

### 1.1. Research Background

Robotic arms equipped with powered end-effectors hold immense potential for advancing automated building construction, mitigating critical challenges such as quality degradation risks arising from reliance on low-skilled labor, an aging workforce, and disruptions due to varied fieldwork demands [1,2,3]. Despite compelling promises, however, multi-functional end-effectors may face difficulties effectively adapting to the highly unstructured task environments typical of building construction sites [4]. Specifically, singular task-specific operations, such as masonry and rebar-tying, underscore the limited versatility of conventional mechanical grippers. These limitations present significant obstacles to the widespread incorporation of robot arms into building processes, as robotic tools are typically designed for predefined tasks in controlled environments. Although automatic tool-changers exist to replace assorted mechanisms, they do not adequately address interoperability between different end-effector products and their adaptivity to unseen conditions. In response to these challenges, multi-functional human-hand-like robot grippers have been developed; electric motors actuate articulated metal finger frames used for grasping various shapes of objects [5,6,7].

However, these rigid-link multi-finger grippers necessitate careful management in unstructured construction tasks due to their intricate mechanisms and the susceptibility to environmental stressors such as temperature fluctuations, exposure to chemicals, dust, moisture, and unexpected impacts. In addition, a large impact load while grasping dynamic objects easily damages both target objects and rigid grippers [8,9]. Frequent work discontinuity due to end-effector malfunction and replacement hinders work processes, requiring constant presence of robotic experts on the site. Additionally, the mechanical complexity due to sensor embedding and sophisticated structure [10,11], the difficulty in handling fragile and arbitrarily shaped objects through pre-programming [12], and the significant risk of human injury from collisions in close distance present further challenges [13,14]. This eventually increases the introduction, maintenance, and operation costs of robotic systems in the construction site [9].

**Figure 1:**
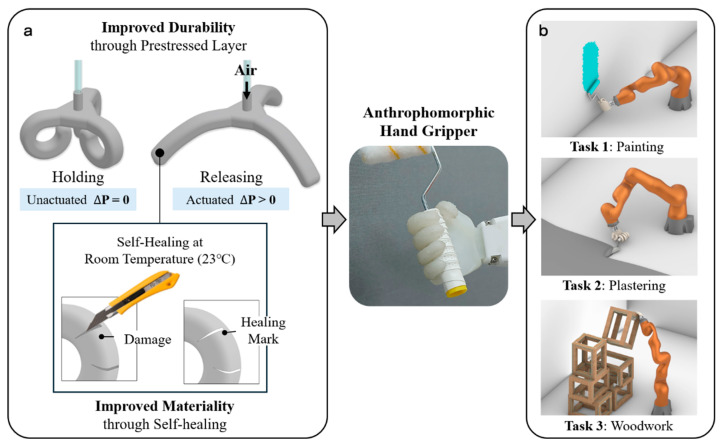
Research concept: (**a**) Two strategies for enhancing the durability of pneumatic actuators: —pre-stressed reversible mechanism (PSRM) and self-healing material (SHM); (**b**) A multifunctional soft hand gripper capable of adapting to various architectural construction tasks.

To address these challenges, this study presents a lightweight, flexible, and multi-functional soft gripper with an improved mechanism and enhanced material composition (Figure 1). Advancing the previous work of Firth et al. [9], we aim to strengthen the operational stability and durability of the anthropomorphic robotic hand for construction applications by refining both the actuation mechanism and the material used for finger structures.

### 1.2. Related Work and Research Gaps

Numerous studies in soft robotics have focused on developing pneumatically actuated robot-arm end-effectors that mimic the geometry and dexterity of the human hand [15,16,17,18,19]. These efforts have led to advanced biomimetic designs that leverage sensor-assisted precision control of internal pressure and morphology. To address the inherent low rigidity of soft robotics, some pioneering grippers often achieved through multi-material 3D printing have been designed with variable stiffness, enhancing their adaptability and functionality [20,21,22]. However, in architecture and construction, ease of integration, material adaptability, and low-cost scalability are often prioritized over high mechanical precision in building production—for example, in on-site brick assembly, plastering, surface finishing, painting, or adaptive tool handling. From this perspective, the multi-functional anthropomorphic gripper developed by Firth et al. [9] proves to be a more practical and efficient solution for achieving building-related objectives through straightforward means. However, while their work demonstrated easily controllable fiber-reinforced finger actuators inspired by human hand anatomy, it has limitations in addressing potential operation failures caused by continuous forced manipulation (inflation) and material fatigue due to constant air compression. Elastomers, commonly used in soft robotics, are susceptible to tearing and air-leaking when subjected to unexpected external forces applied to their surfaces. Robot arms used in architectural fabrication and building construction are more often tasked with holding irregular tools and rough materials for extended periods rather than performing quick pick-and-place actions, which undermines grippers’ durability and reliability in real-world applications.

To overcome these limitations, we leverage (i) the pre-stressed reversible mechanism (PSRM) and (ii) a novel self-healing material (SHM) developed by the authors. As demonstrated in Pal et al. [23], the hyper-elastic PSRM draws inspiration from the elastic energy storage mechanisms found in the animal musculoskeletal systems and tendons, aiming to minimize expansion time and enhance behavioral stability during inflating [23,24,25,26]. The PSRM-integrated actuator, unlike conventional pneumatic soft robots, is capable of grasping objects while in a deflated state without the need for external pressure. It maintains a bent shape and deforms when inflated, quickly recovers its original shape once the pressure is released. This innovative approach helps to improve the work efficiency and operational reliability of soft robot grippers, particularly in applications where prolonged holding of an object is required. On the other hand, the utilization of SHM, a type of smart polymer, can improve the functionality of the hand gripper by enabling self-repair of torn sections, akin to the regenerative properties of human skin [27,28]. Studies have shown that when a damaged (ripped) polymer undergoes proper heating, and the Diels–Alder reaction [20,29,30] occurs, it can regain mechanical strength comparable to its pre-damaged state, even when completely severed. However, while consistent repair is theoretically achievable, and Terryn et al. [29] demonstrate the restored elasticity of the SHM elastomer after being cut, maintaining the chemical stability of SHM requires additional heating and cooling processes to ensure robust actuation spontaneity. To tackle this issue, a novel skin-like polymer substrate is developed through a synthesis of polyborosiloxane (PBS) and Ecoflex™ [31]. This new SHM, the PBS–Ecoflex hybrid polymer (PEHP), exhibits autonomous self-healing capabilities, allowing it to restore its original properties at room temperature without requiring any additional physical or chemical intervention for repair. It showcases durable self-healing capability that lasts over six months, effectively mitigating chemical instability within the PBS matrix and bolstering material resilience against environmental stressors such as exposure to extreme temperature, water immersion, strong acids/alkaline, and many other chemicals. By incorporating this skin-like self-healing material, we significantly improve the durability and functionality of our soft robot grippers in architectural applications, delaying failures caused by external damage and localized degradation from repeated use, ultimately preventing work interruptions.

### 1.3. Study Objectives

We develop a versatile robot-arm end-effector suitable for enhancing its workability and durability in unstructured building construction environments. The major thrusts of this study are summarized as follows:

- Design of a biomimetic soft-robot mechanism adaptive to building construction: Development of an anthropomorphic robotic gripper specialized for long-duration holding tasks in construction, including tool grasping, stabilization of irregular objects, and adaptation to dynamic on-site environments.
- Integration of a novel self-healing material: Utilization of advanced self-healing materials to fabricate soft robotic fingers, ensuring durability and the ability to self-repair under mechanical stress or damage.
- Performance evaluation and practical demonstration: Testing of the robotic hand’s performance and validation of its utility in executing building construction tasks.

## 2. Materials and Methods

### 2.1. SHM Synthesis: Physical Polymer Cross-Linking

**Figure 2:**
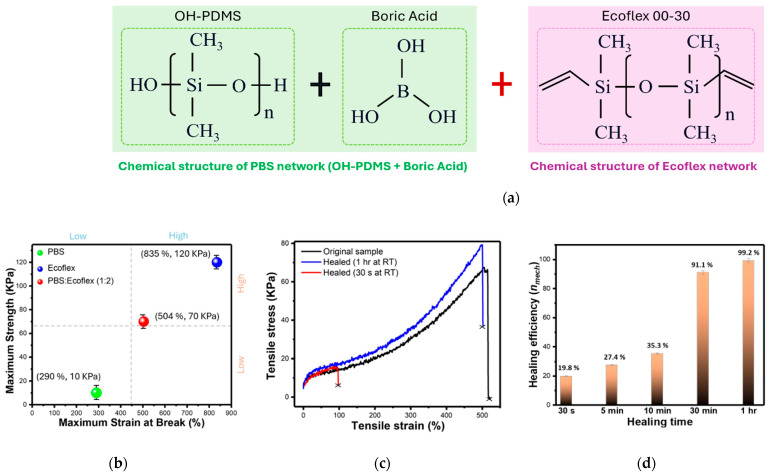
Hybrid polymerization of PBS: Ecoflex (silicone) substrate and its self-healing performance: (**a**) Illustration of the chemical-physical structure of PEHP and its double-network hybrid polymerization; (**b**) Mechanical properties of bare PBS, Ecoflex™, and PEHP; (**c**) Stress–strain curves of PEHP healed at room temperature, showing that substrate’s stretchability approximates its original (pre-damage) state with longer healing durations; and (**d**) Self-healing efficiency, expressed as the recovery of maximum mechanical strain. Reproduced with permission from [31]. © 2023, Elsevier.

**Figure A1:**
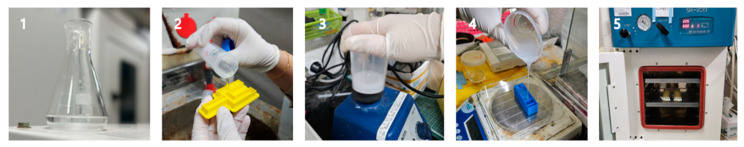
PEHP curing process in 3D geometries.

The SHM used in this work is a unique polymer exhibiting notable self-healing and elastic properties suitable for the study purpose. The SHM was fabricated using a facile co-curing polymer synthesis method, which involved silicone vulcanization of PBS and Ecoflex™ (a platinum-cured polydimethylsiloxane (PDMS) rubber, Smooth-On, Inc., Macungie, PA, USA) (see Appendix A, Figure A1). As illustrated in Figure 2a, PBS retains chemical units with unreacted Si–OH or B–OH groups, whereas Smooth-On Ecoflex™ is a chemically inert, highly cross-linked Si–O–Si elastomer. Despite the inherent challenges in achieving cross-linking between the two distinct systems, our experiments revealed that physical entanglement can occur when they are thoroughly mixed and cured together in their uncured (liquid) states, leading to the formation of interpenetrating polymer networks. It can be facilitated by uniform dispersion of the two polymers and the high density of Si–O–Si backbones present in both materials [32]. Additionally, during curing, residual Si–OH groups in PBS likely react with Si–H groups from the Ecoflex™ Part B, forming covalent Si–O–Si bridges at the PBS-Ecoflex interface. The physical cross-linking via topological entanglement may also be enhanced by hydrogen bonding and dative bonding between boron and oxygen atoms within the Si–O networks.

These reactions initiate immediately upon mixing the PBS and Ecoflex polymer solutions, with heating at 60 °C for 24 h required to complete curing. After curing, the resulting PEHP solidifies in the exact size and shape of the mold (it can be further trimmed as needed). Although this synthetic mechanism is not a standard or fully characterized reaction, the mechanical self-healing performance and properties of PEHP were identified and evaluated in our previous study [31] using a planar configuration (20 × 10 × 5 mm substrate). The healing efficiency was demonstrated by a 99.2% recovery of the sample’s maximum strain within one hour (Figure 2d).

### 2.2. Biomimetic Design Concept and Driving Mechanism

**Figure A2:**
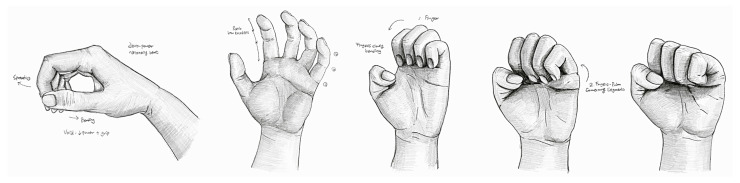

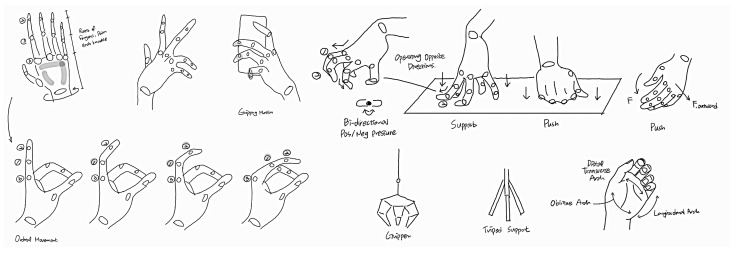
Preliminary architectural study to identify biomechanical mechanisms responsible for different types of human hand movements.

Our gripper design emulates the anatomical properties of the human hand, utilizing pre-stressed driving mechanisms to effectively prototype and evaluate object grasping performance. Through comprehensive examination of hand gestures (Appendix B Figure A2), we observed that the natural resting position of the human hand forms a gentle curve when suspended without exertion of force. This position can be modified with muscular engagement to achieve either full extension or complete flexion from this relaxed state. The hollow cavity naturally formed between flexed fingers and the palm creates an adaptable void space that enables stable object retention from multiple angles with minimal force application. By incorporating a pre-stressed design concept, we replicated this void space by engineering a semicircular bent configuration in the linear actuator.

Based on conceptual knowledge, various 3D geometries of PEHP (e.g., fingers and palm structures) were prototyped, integrating PSRM and multi-layer fabrication techniques. Implementation of the PSRM enables finger abduction when pneumatic pressure is applied. In fact, our analysis revealed that the three phalanges of each human finger undergo sequential folding and unfolding motions during natural hand movements. We enhanced this biomimetic property by strategically reconfiguring the air chamber within the pneumatic networks (pneu-nets) of the finger actuator, thereby augmenting the sequential movement capabilities of the actuator system [33]. The palm of the human hand also plays a crucial role in distributing loads and controlling and manipulating multiple objects. Three arches that run in different directions on the anterior surface create concavity that aids in pre-shaping and grasping [34]. Similarly, our palm prototype model implements the longitudinal arch out of these three arches to support stable grasping. This biomimetic feature is achieved through the contraction of an elastomer upon the removal of the previously applied external force.

### 2.3. Experimental Procedure

**Figure 3:**
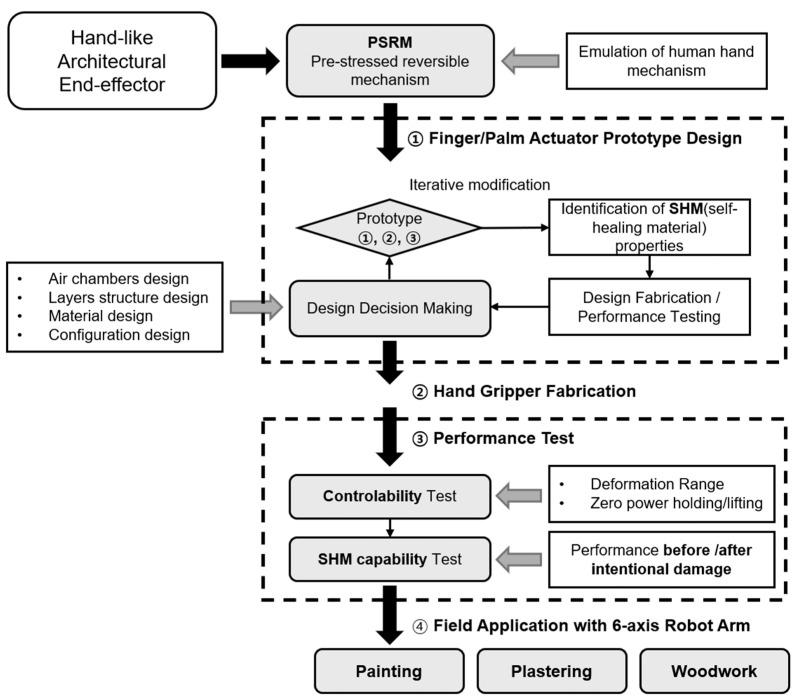
Methodological procedures.

Our study followed a structured experiment approach as illustrated in Figure 3. We began with a comprehensive literature review on effective pre-stressed soft actuator design, followed by iterative design modifications based on material property characterization and preliminary laboratory testing. This process ensured proof-of-concept validation for the actuator’s suitability in lightweight construction tasks. Critical variables including air chamber configuration, layer structures, and pneumatic networks were systematically adjusted (①). Upon finalization, the actuators were assembled into functional units (②) and subsequently tested for zero-power holding capability and self-healing performance both before and after induced damage (③). As a culmination of our research, the fabricated end-effector was integrated with an industrial robot arm to demonstrate practical application through robotic construction tasks (④). This research represents a multi-disciplinary design approach that integrates material engineering principles with biomimicry concepts, specifically targeted toward architectural robotic hand tools. While the presented end-effector has not yet reached commercial availability, our investigation demonstrates the significant potential benefits of collaborative interaction between non-professional workers and construction robots in architectural applications.

### 2.4. Design and Fabrication of Gripper Fingers

#### 2.4.1. Finger Design: Proof-of-Concept

Three different geometries of PEHP-enabled anthropomorphic gripper fingers were prototyped as summarized in Table 1. Each finger gripper design was developed to implement targeted motions and leverage the characteristics of PEHP, aiming to improve actuator durability by protecting the most vulnerable upper expansion part where constant air pressure is stressed. In our material testing, fully cured PEHP demonstrated compatibility with other elastomers and silicone adhesives, while the rheological properties of PBS exhibited slower shape restoration. Notably, PEHP in its liquid state (immediately after mixing and before heat treatment curing) demonstrated strong adhesion properties to solid or cured Ecoflex layers, facilitating robust multi-material integration. To compensate for the viscoelastic properties of PBS, the bottom layer of the PSRM structure incorporated silicone elastomer, which enhances shape retention and restoration capabilities. The evolution of our finger design progressed by pre-examining three feasible distinct prototypes:

- Type 1: A dual-layer elastomer configuration where the top layer contains linearly connected air chambers adhered to a pre-stretched non-SHM elastomer bottom layer. This design facilitates programmable actuation through adjustments to the direction and magnitude of the stretching force.
- Type 2: An exploration of double pneu-net PEHP layers for the upper section to augment bending capability and enhance object holding through inflation of the top layer, mimicking the bidirectional motion characteristic of human fingers. However, the inherent weak stiffness of PEHP due to PBS content and inadequate reaction following complete solidification resulted in complex air chamber arrangements that hindered rapid shape recovery during deflation and complicated the manufacturing process.
- Type 3: Our optimized final design features a thickened PEHP layer with simplified chamber geometries specifically engineered to replicate human finger actions. The incorporation of glass wool fiber reinforcement mimics elastic ligaments and significantly enhances the stiffness of the PEHP layer. This fiber enhancement effectively guides directional bending of the finger, resulting in improved performance by minimizing unintended inflation patterns.

#### 2.4.2. Fingers Fabrication

**Figure 4:**
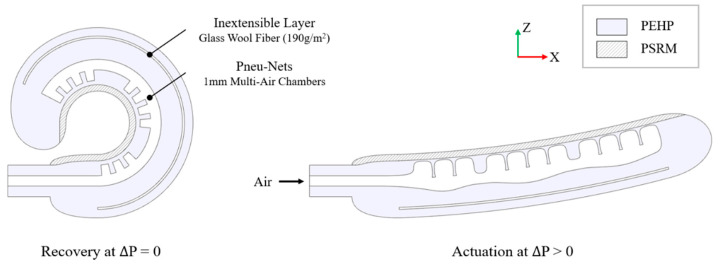
The driving mechanism of the type 3 finger actuator.

**Figure 5:**
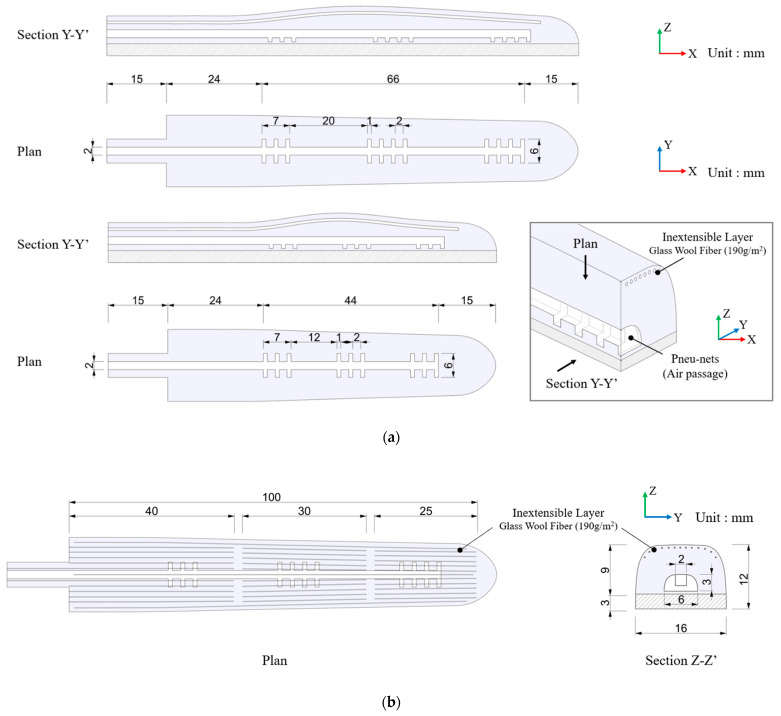
Finger design plans and cross-sections: (**a**) The long actuator module is applied to the index, middle, and ring fingers, while the short module is used for the thumb and little finger; (**b**) Reinforcement strategy of embedding glass wool fibers, indicating their location and length.

As depicted in Figure 4, our design was refined based on Type 3, incorporating glass wool fiber (190 g/m^2^) embedded in the upper part of the top layer. Fiber reinforcing effectively constrained unnecessary inflation, resulting in improved rigidity and shape maintenance. Upon air supply, neighboring chambers properly expanded and aided in spreading the fingers apart with minimal air usage, thus preventing stress over the layer surface. Figure 5a illustrates two finger fabrications, each with varying lengths: one with a length of 120 mm and the other with 98 mm. In these variations, all five fingers were completed for the gripper. The bottom layer of the gripper was crafted from a flat-shaped Ecoflex™ 50 sheet with a thickness of 3 mm. Prior to assembly, it was axially stretched to twice its original length, ensuring ample elastic energy storage for shaping into a pre-stressed state. The individual grippers were integrated into the palm structure. During practical operation, this configuration maximizes contact area with target objects, closely resembling the grip mechanics of a clenched human hand. The rolled finger design enables secure wrapping around objects of various geometries and dimensions without requiring additional pneumatic pressure or power input. This versatility provides efficient gripping capability across diverse task requirements. Figure 5b details dimensional specifications for both sectional and plan views, along with the strategic arrangement of reinforcing fibers within the gripper structure.

**Figure 6:**
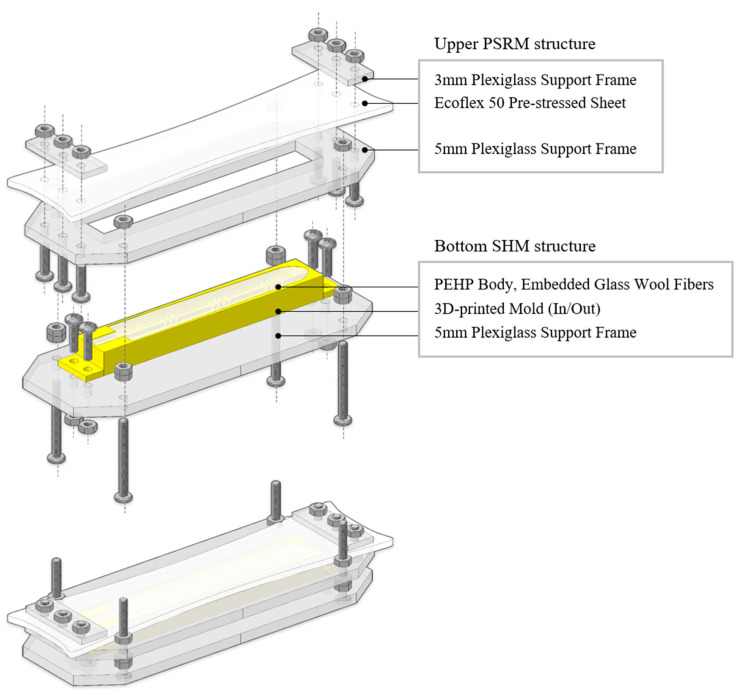
Finger actuator fabrication: Support frame details and assembly method.

**Figure 7:**
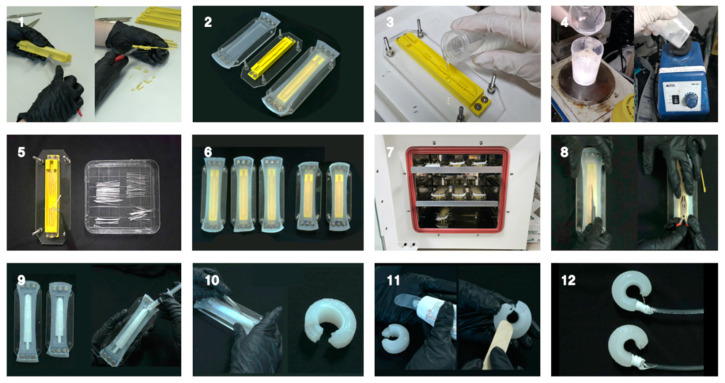
Fabrication process (finger actuators): (1) Fabrication of the inner and outer mold of the finger actuator; (2) Upper PSRM structure; (3) Polyvinylpyrrolidone (PVP) poured into the molds; (4) Mixing of PBS and Ecoflex™ 30 in liquid state; (5) Reinforcement with glass wool fibers; (6) Assembly of PEHP mixtures using support frames; (7) Curing at 60 °C for 24 h in a digital electric furnace; (8) Removal of the inner mold through a small incision at the bottom; (9) Separation of the upper frame; (10) Trimming of the outer contours; (11) Sealing of the incision from Step 8 using Ecoflex™ 30; and (12) Material comparison of the completed fingers: Ecoflex 50™ (top) and SHM (bottom).

In Figure 6, comprehensive manufacturing specifications for finger components are presented. A total of 72 mL PEHP solution was used to fabricate all five fingers, with 48 mL allocated for the longer fingers and 24 mL for the shorter ones. The manufacturing process began with stretching and firmly fixing the precured Ecoflex layer onto a 5 mm-thick plexiglass frame. This was then tightly assembled with a complementary support frame containing a 3D-printed PEHP mold, ensuring complete interface adhesion between the two layers during the PEHP curing process, which was conducted at 60 °C for 24 h. Following the curing phase, the inner mold for the air channels was carefully extracted through a small incision created on the underside of the assembly. The resultant small cavity, positioned opposite the air inlet, was meticulously sealed using Sil-poxy™, a room-temperature-vulcanizing (RTV) silicone (Smooth-On, Inc., Macungie, PA). Subsequently, the actuator body was precisely separated from the prestressed layer along the predetermined perimeter outline. To complete the assembly, as shown in Figure 7, a 1 mm-thick elastomer sheet was affixed over the incision on the interior surface of the circularly rolled actuator using the adhesive.

### 2.5. Design and Fabrication of the Palm

#### 2.5.1. Palm Design

**Figure 8:**
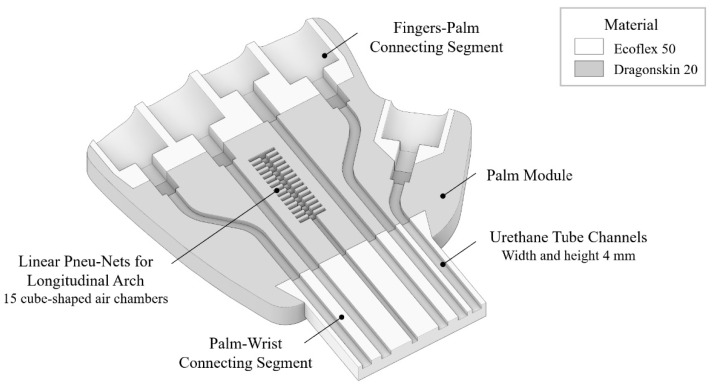
Design of the palm actuator: Materials, structure, and internal air chambers.

**Figure 9:**
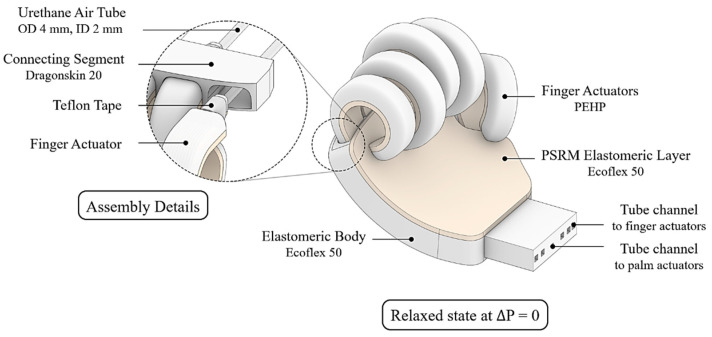
Assembly of the finger modules and palm actuator.

The palm actuator serves as the central connecting element for the finger components (Figure 8 and Figure 9). By incorporating a gentle curvature that complements the finger flexion, an internal hollow space is formed, effectively mimicking the longitudinal arch configuration of the human hand. To enable precise individual finger control, urethane tubing with an outer diameter of 4 mm and an inner diameter of 2 mm was integrated into the palm structure to facilitate pneumatic supply distribution.

Within the palm, a series of 15 cube-shaped air chambers, each precisely dimensioned at 9 mm, 1 mm, and 4 mm in width, length, and height, respectively, were arranged in a repetitive pattern between the pneumatic tube channels connecting to the middle and fourth fingers. The controlled inflation of these chambers serves to increase the curvature of the palm actuator, thereby enhancing its grasping capability and biomimetic function. As depicted in Figure 8, the palm consists of a main body and three connecting components. These joint sections were fabricated using Smooth-On Dragonskin™ 20, a higher durometer silicone elastomer, to ensure secure and durable fixation of the actuator elements. The main body incorporates pneu-nets constructed from Ecoflex 50, facilitating a more naturally flexed hand configuration when subjected to pre-stress conditions. Each component was specifically designed for independent fabrication, with final assembly conducted after complete silicone curing, as demonstrated in Figure 9.

#### 2.5.2. Palm Fabrication

**Figure A3:**
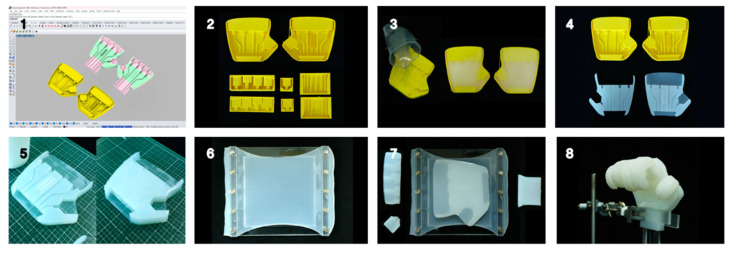
Fabrication process of the palm actuator.

The fabrication of the palm actuator involved multiple steps, starting with mixing and pouring liquid-state silicone into a 3D-printed mold, followed by a four-hour curing process at room temperature. Due to the intricate design of the palm—featuring structural elements such as tube channels and interconnected air chambers—the actuator was cast in two separate halves at an optimal division point. After demolding, the two halves were carefully aligned and bonded together to complete the assembly, ensuring structural integrity and airtightness (Appendix C Figure A3).

## 3. Results and Discussion

### 3.1. PEHP Material Behavior

**Figure 10:**
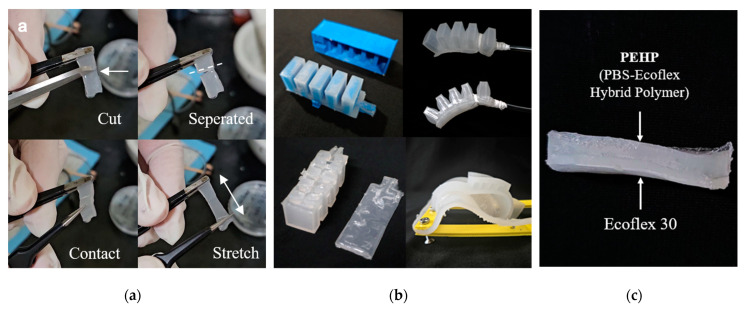
Self-healing tests of PEHP and troubleshooting during actuator fabrication: (**a**) Recovery of elasticity at room temperature following complete incision; (**b**) Integration failure between PEHP and a chemically similar silicone material after curing; and (**c**) Successful adhesion between PEHP and silicone elastomer achieved through proper bonding techniques.

Initial investigations focused on SHM performance and troubleshooting in actuator manufacturing. Consistent with prior work [31], the synthesized PEHP sample exhibited excellent self-healing performance at room temperature (Figure 10a). Repeated material tests characterized PEHP’s suitability for complex, finger-like geometries, demonstrating stable elastic energy storage with minimal degradation. To evaluate its integration potential, the upper segment of a simple actuator with a pleated morphology and five internal air chambers was fabricated and bonded to another SHM sheet using liquid-state composites, silicone, and silicone adhesives. However, despite ensuring uniform curing conditions and adhering actuator components with PEHP, the materials did not fully harden, resulting in air leakage at the joint surfaces. Similar adhesion challenges arose when combining PEHP with elastomer, using RTV silicone adhesive, as PEHP exhibited poor compatibility with cured silicone materials (Figure 10b). Additionally, the viscoelastic behavior of PBS exhibited a slow recovery time exceeding ten seconds after deformation, underscoring its limitations for pre-stressed structures. During manufacturing, PEHP curing began immediately upon mixing, necessitating prompt pouring into molds to ensure complete solidification. Subsequent experiments revealed that PEHP could be effectively integrated with solidified silicone elastomers when liquid PEHP was applied before thermal treatment, ensuring thorough contact (Figure 10c). This approach facilitated the fabrication of stable pre-stressed structures with rapid recovery and sufficient shape-retaining rigidity. The assembled components were successfully validated as a pneumatic soft actuator, demonstrating airtight functionality through seamless adhesion between the elastomer sheet and the PEHP component.

### 3.2. Proof-of-Concept Fabrication

**Figure 11:**
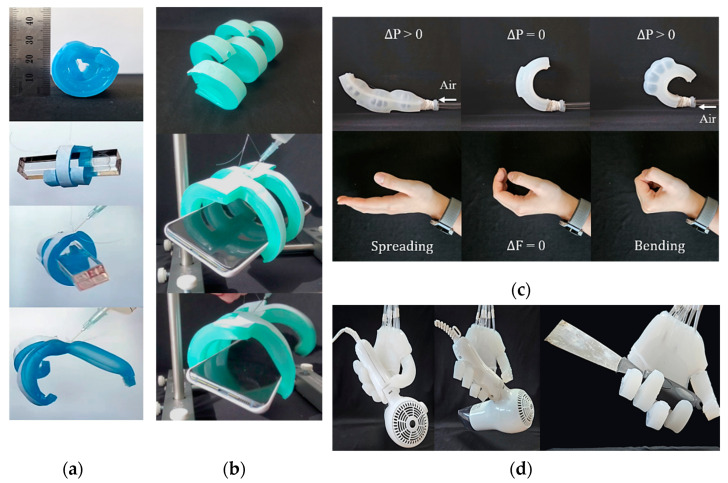
Proof of PSRM concepts in soft grippers: (**a**) Three legs holding and releasing objects; (**b**) Prototype model grasping a smartphone; (**c**) Type 2 gripper exhibiting stable bi-directional movement through inflation; and (**d**) Soft robot hand effectively grasping geometrically complex objects.

Proof-of-concept prototypes were fabricated to validate the PSRM finger design. As exhibited in Figure 11a,b, the initial flexed gripper legs—designed based on Pal et al. [24] and weighing only 40 g—demonstrated effective object lifting and retention. By incorporating a pre-stressed layer, the multi-leg gripper securely held a smartphone weighing about 190 g, achieving a payload-to-weight ratio of nearly 5:1. During operation, the gripper released objects by unfolding its legs, triggered by an air injection of ~75 mL. The second anthropomorphic finger prototype (Figure 11c,d) was capable of hyperextension and flexion, closely replicating natural hand movements. However, pre-stressed fingers with a half-circular bending configuration exhibited unreliable object retention and lifting without supplemental air input, largely due to the motion moment. Furthermore, our experiments revealed persistent challenges, including recurrent layer detachment during the fabrication of the tri-layer design (Type 2) and air leakage stemming from insufficient interlayer sealing.

### 3.3. Mechanical and Self-Healing Performance

**Figure 12:**
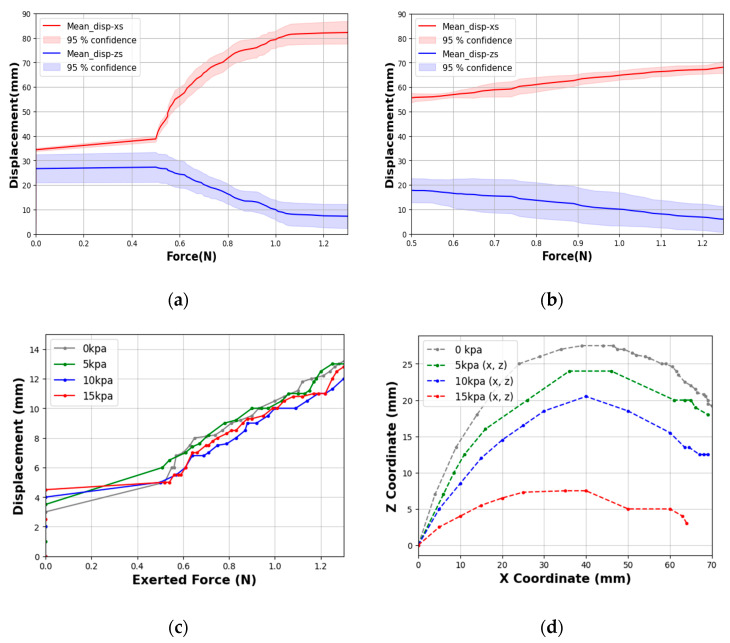
Mechanical response of a single finger (fiber-reinforced). (**a**) Fingertip displacement in zero-pressure state under tensile loading. (**b**) Recovery phase displacement under zero-pressure state. (**c**) Shape retention capability under varying internal pressure. (**d**) Deformation trajectories under different pressure conditions.

The PSRM-induced gripping tip force of a single finger segment was measured by securing the air inlet in a fixed chuck. Figure 12a presents the force-displacement curves generated by applying a continuous external tensile force using a force gauge (EW-25C digital force meter, EASTONTECH China) via a nylon thread attached to the gripper tip, which was used to unfold the finger. Figure 12b displays the force–displacement profiles during the shape recovery phase, during which the gripper returns to its original rolled configuration upon force removal. All tests were repeated five times under identical conditions, with the average displacement trends on the X- and Z-axes (Figure 4) recorded. The 95% confidence interval around the mean displacement values for both axes indicated high reproducibility across trials, confirming the reliability of the gripper’s response.

These results show that the displacement peaked at approximately 1.0 N, when the finger was stretched. We further evaluated the gripper’s pressure-assisted shape retention capability by applying unfolding tensile forces at the gripper center via a nylon thread under four discrete input air pressures (0, 5, 10, and 15 kPa), while measuring the force-displacement relationship along the X-axis (Figure 12c). Notably, among the four variants, the system exhibited minimal deformation relative to external force at 10 kPa internal pressure after application of 0.6 N external force, establishing this as the pressure condition suitable for deformation resistance. At the maximum applied force of 1.2 N, the displacement measured 11 mm under 10 kPa internal pressure—significantly lower than the 13 mm observed at 0 kPa. As external force increased, performance at 15 kPa became comparable, indicating that higher internal pressures effectively reduce deformation but with diminishing returns beyond 10 kPa. Figure 12d shows the gripper’s fingertip displacement profiles across varying internal pressures, further demonstrating pressure’s role in mitigating deformation. Notably, at 15 kPa, vertical displacement remained below 5 mm, versus 15 mm in the zero-pressure state. These results corroborate prior studies [35,36], confirming that both 10 and 15 kPa offer superior shape retention.

The 10 kPa condition demonstrates an optimal balance, effectively minimizing displacement while preserving adaptability to external forces. In contrast, while 15 kPa provides superior deformation suppression, it substantially compromises flexibility, potentially limiting practical utility. These findings demonstrate that internal pressure can be precisely adjusted to achieve application-specific rigidity-flexibility trade-offs. Compared to conventional soft-robot grippers actuated by inflation (Table 2), our fiber-reinforced PSRM design maintains comparable tip forces and resistance even in a zero-pressure state. While typical air-inflated single grippers with semi-circular configurations exhibit grasping forces of 0.17–1.6 N and maximum payloads below 1.25 kg [36,37,38,39,40,41], the PSRM gripper achieves zero-power holding with merely ~10 mm displacement under 1.0 N external force. This capability enables reliable operation without continuous air supply or chamber inflation, offering a significantly more energy-efficient alternative to conventional pneumatic approaches.

**Figure 13:**
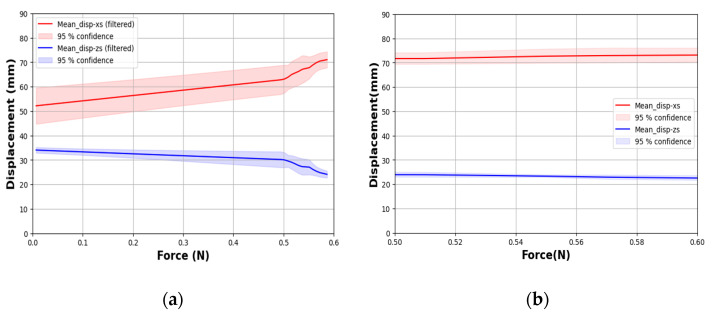
Mechanical response of a single pure PEHP finger (no fiber). (**a**) Fingertip displacement in zero-pressure state under tensile testing. (**b**) Recovery phase.

On the other hand, as shown in Figure 13, the displacement capacity of our PEHP finger (no fiber) under tensile loading is comparable or superior to several elastomeric designs (Table 2). Our design achieves ~0.6 N of reaction force at only ~70 mm of displacement, highlighting its high strain sensitivity under sub-Newton forces. Unlike pneumatic actuation, this purely mechanical response is generated from external tensile force without internal air pressure, allowing us to characterize the intrinsic structural flexibility of the elastomeric fingers under passive conditions.

**Figure 14:**
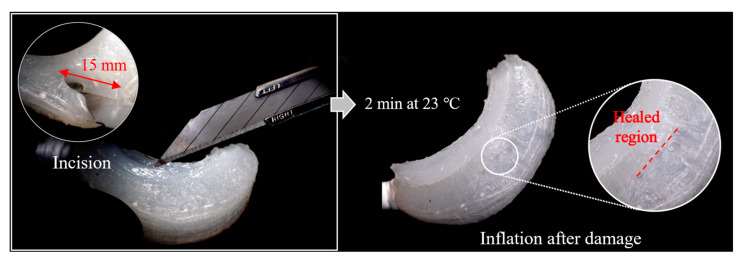
Self-healing capability of the finger: A 15 mm-wide, 8 mm-deep incision made using a cutter.

Meanwhile, the self-healing capability (about 99% maximum strain recovery after an hour [31]) was observed within the finger design. An incision, 8 mm deep and 15 mm wide, was intentionally made on one side of the actuator, resulting in complete damage to the internal air chamber. The actuator was then left undisturbed at room temperature for two minutes to allow for healing. As depicted in Figure 14, the damaged region showed substantial recovery, with only a minor healing mark remaining on the surface. Moreover, the actuator exhibited identical deformation characteristics as observed prior to the incision, with no evidence of air leakage, even after undergoing over 10 cycles of repeated injection testing. These findings provide strong evidence for the effectiveness of self-healing materials in enhancing the durability and long-term maintenance of soft matter-based systems, thereby reducing the need for frequent replacements (Supplementary Video S1).

**Figure 15:**
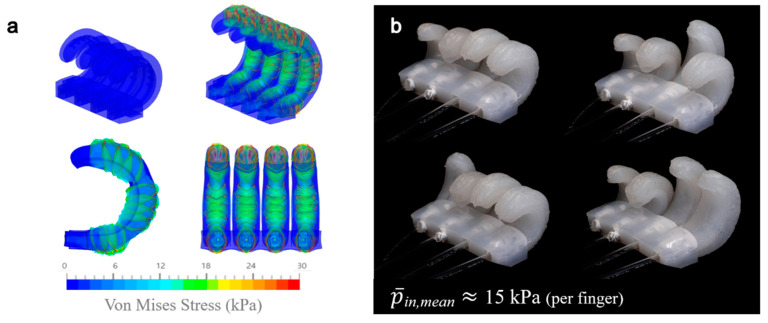
Fingers in motion: (**a**) The nonlinear deformation of the actuator was predicted using finite element analysis in SimScale^®^ at an inlet pressure 10 kPa; (**b**) Finger actuators were embedded in a finger-palm connecting segment and individually controlled at an average inlet pressure of 15 kPa (measured using a Vernier^®^ GDX-GP pressure sensor, Beaverton, OR, USA).

**Figure A4:**
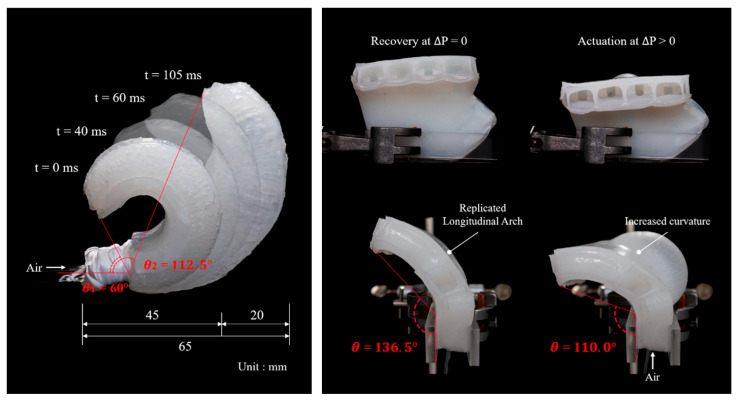
Deformation behavior of the pre-stressed finger (left) and palm actuator (right).

The actuator was connected to a 60 mL syringe through a urethane tube with an outer diameter of 4 mm, which was securely sealed using 12 mm wide Teflon tape. Approximately 40 mL of air was injected into each finger module to facilitate its expansion. Initially, the actuator was in a deactivated state, characterized by a stable circular shape between the air inlet and the fingertip, with an approximate angle of 60°. Upon air injection, the actuators gradually expanded to an angle of 112° (Appendix D Figure A4). Notably, these actuators rapidly returned to their original configuration within one second after deflation. The finger actuators are capable of operating either individually or in groups, depending on the configuration of the air inlets. The experimental validation shows strong agreement with hyperelastic finite element simulations (Figure 15, SimScale GmbH) using a Neo-Hookean model with the following parameters: C_10_ = 1.15 × 10^−2^ MPa (PEHP layer) and 1.38 × 10^−2^ MPa (PSRM layer), D_1_ = 1 × 10^−5^ Pa^−1^, and ρ = 1000 kg/m^3^. Furthermore, the palm module, which incorporates a pre-stressed layer on the inward side of the hand, exhibits a gentle curve in its initial state. Upon air injection into a linearly arranged air chamber located in the center, the palm undergoes additional bending. With an air supply of 60 mL, the palm reaches an angle approaching 90°, facilitating the formation of a more stable circular cavity when integrated with finger actuators. This design enhances the overall grasping capabilities, improving the effectiveness of the gripping mechanism (Appendix D Figure A4).

### 3.4. Performance of Soft Hand Gripper as Versatile End-Effector

**Figure 16:**
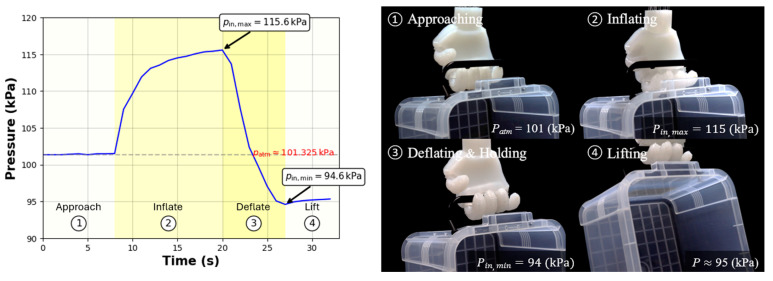
Pressure profile of the zero-power holding and lifting a 0.6 kg plastic box. The hand gripper maintains a stable grasp with no external power supply. Measured inlet pressures (including min, max, and atmospheric pressure) are indicated.

**Figure A5:**
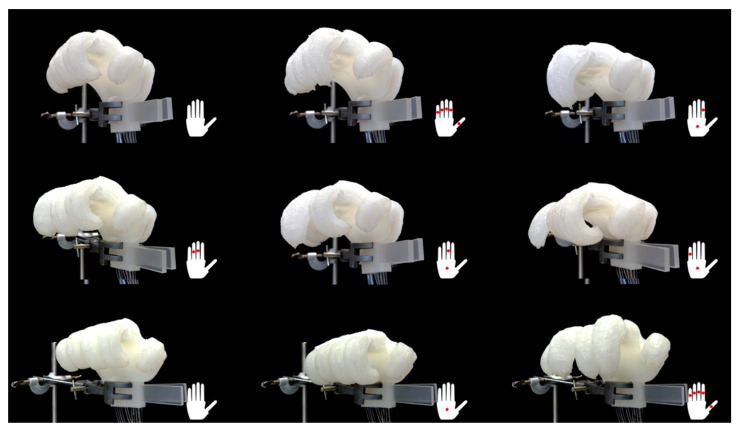
Deformation patterns generated by various actuation combinations: Red dots indicate the actuated regions responsible for each movement.

The fully assembled self-healing hand gripper measures 110 mm in width, 100 mm in height, and 18 mm in thickness, closely resembling the natural resting shape of a human hand. The gripper weighs only 300 g, which is significantly lighter than rigid robotic hand grippers of comparable size, typically weighing over 1 kg. Equipped with both finger and palm actuators, the gripper enables agile and adaptable movements (see Appendix E Figure A5). Figure 16 illustrates the zero-power holding mechanism: when inflated, the gripper flexes towards the handle of a tool or object, achieving a firm grasp. Upon deflation, it passively returns to its original configuration, allowing it to securely hold and lift objects without requiring continuous external power input.

**Figure 17:**
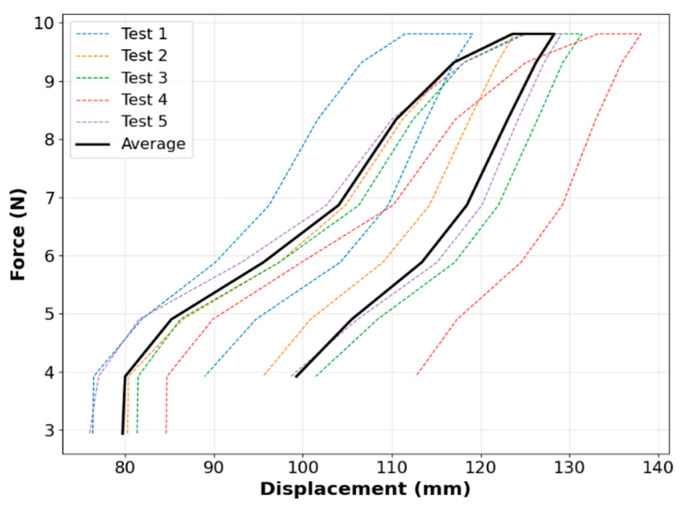
Force–displacement test result: loading and unloading cycle.

To simulate general holding resistance of the hand, a tensile test was performed, using a universal testing machine (UTM, LR-C003, 0–20 kN, Lonroy Equipment Co., Ltd., Dongguan, China), as shown in Figure 17. A cylindrical object gripped by the hand was cyclically pulled and released along the grasp direction. The maximum reactive holding force before slippage was measured at ~13.7 N, while the peak tensile force of 9.8~10 N was applied to characterize the hand’s force-displacement behavior. Variations observed across the five repeated trials likely reflect intrinsic deformation variability and uncontrollable experimental uncertainties, such as differences in object alignment or finger contact conditions. The nonlinear increase in force with displacement indicates progressive stiffening, a characteristic of elastomer-based actuators.

The estimated stiffness of ~0.33 N/mm is marginally higher than that of several existing soft robotic grippers [37,38,39,40,41,42], which typically exhibit values in the range of 0.2–0.35 N/mm, including both research prototypes and commercial products. Moreover, the test revealed noticeable hysteresis between loading and unloading phases, attributed to internal viscoelastic damping, friction between contact surfaces, and the delayed elastic recovery of the soft elastomer material. This hysteretic behavior is intrinsic to soft robotic actuators and reflects energy loss during cyclic actuation, which can influence the repeatability and control accuracy of gripping performance. The relatively consistent envelope across trials suggests that the hand maintains a robust and compliant grip under varying load conditions.

**Figure 18:**
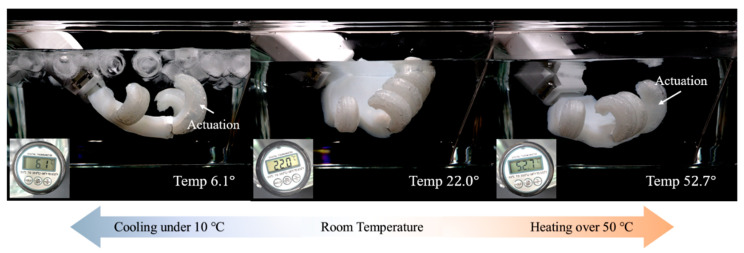
Gripper operation in an underwater environment across various temperature ranges.

**Figure 19:**
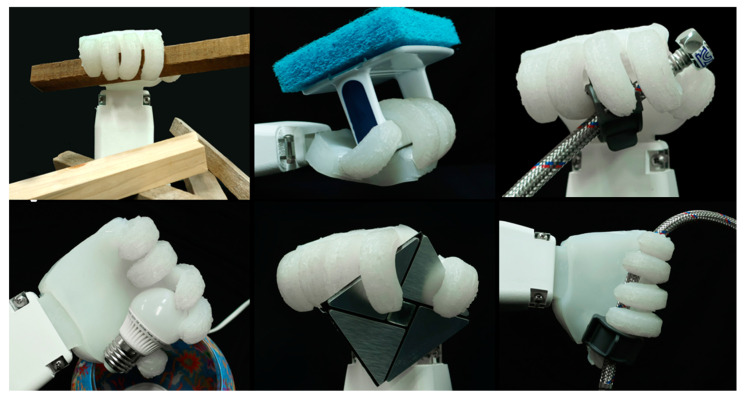
Empirical evaluation of the developed soft hand in grasping irregularly shaped objects.

Nevertheless, due to its inherent softness, this hand is not compatible with heavy-duty tasks such as earthwork, foundation work, or reinforced concrete operations, which require substantial power generation. However, it is well suited for various finishing tasks, where it can work in conjunction with tools that have handles, collaborating closely with human operators. The gripper exhibits rubber-like elasticity and contains no internal circuits, making it safe for use in high-temperature and high-voltage environments. It is also suitable for direct contact with water-containing materials, including those used in plumbing, tiling, and plastering work. The gripper demonstrated consistent performance in underwater environments and across a temperature range of 6 °C to 52 °C during repeated use (Figure 18). Furthermore, it reliably grasped a variety of hand tools and building materials—including lumber, cleaning brushes, paint rollers, faucets, hoses, light bulbs, and decorative tiles—without the need for pneumatic power, additional sensors, image scanning, or pre-programmed control. This highlights its versatility and suitability for a wide range of applications in finishing work (Figure 19).

### 3.5. Field Tests: Robotic Arm Operation

**Figure 20:**
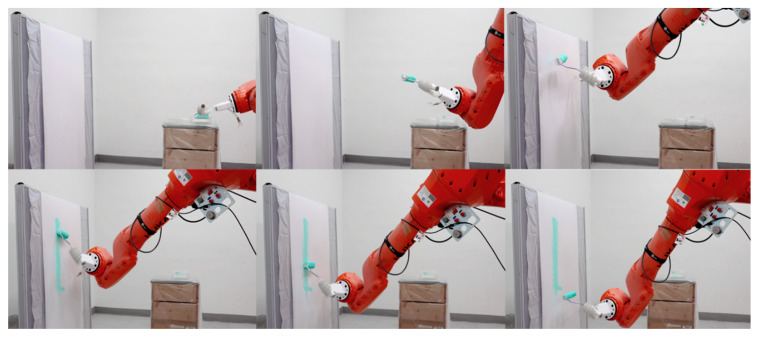
Validation task 1: Performing a paintbrush operation.

To demonstrate the dexterity and functional control of the anthropomorphic robotic hand in robot-assisted architectural and building practice, we evaluated its performance across three representative fieldwork scenarios: painting, plastering, and timber assembly. The gripper tool was integrated with industrial robot arms (KUKA KR50 R2500 and ABB IRB1600). A robotic painting experiment was conducted to validate its utility and propose a lightweight robotic construction system. The hand was mounted on a 3D-printed base fabricated using a MakerBot Replicator (1.75 mm polylactic acid (PLA) filament). A 2-mm plexiglass chuck securely fastened the gripper to prevent rotation or displacement during operation, while urethane tubes connected to the gripper were neatly organized within the platform. The painting task was programmed in the RoboDK environment to control the robot arm, and the program was tested in simulation for gripping, approach, vertical movement, and return sequences. During the operation, the gripper reliably held a 10 cm-wide paint roller with a straight handle, maintaining stability at various angles without the need for air intervention. It performed precise, repetitive painting motions by tilting upward while synchronizing with the robot arm’s movements, effectively covering the partition with paint (Figure 20, Supplementary Video S5). This demonstrates the efficient zero-power holding operation in an elementary painting task.

**Figure 21:**
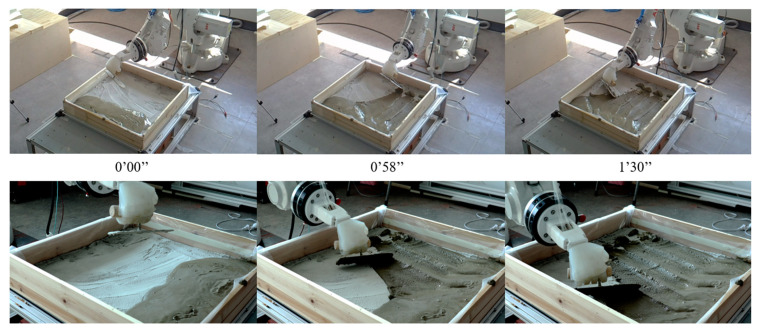
Validation task 2: Floor cement spreading and plastering. It successfully grasps and operates a 200g trowel without an external power supply.

**Figure 22:**
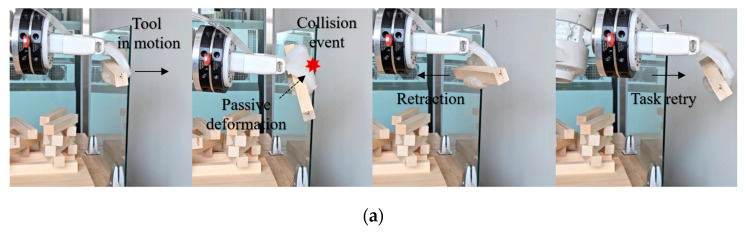

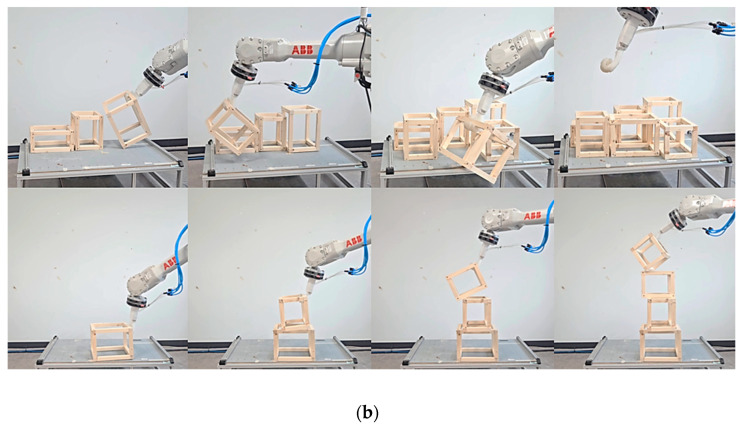
Validation task 3: (**a**) It effectively absorbs impact loads, enabling safe interaction with fragile objects in construction; (**b**) Woodworking task involving the stacking of timber boxes.

Additionally, cement plastering (Figure 21) and the picking and placing of timber boxes (Figure 22) were carried out using an ABB IRB 2600 robot arm. Unlike mechanical grippers, the soft hand gripper incorporates an efficient energy regulation mechanism that absorbs external impact loads. It demonstrates the ability to securely grip and adapt to dynamic targets with varying shapes, masses, scales, and textures, minimizing the impact moment between the gripper and the target objects. This capability reduces the risk of malfunctions during operation by allowing the gripper to flexibly restore its shape, preventing potential damage while maintaining a secure grasp on the objects.

### 3.6. Limitations and Further Challenges

Although the developed tool is effective for dynamic handling tasks related to construction, several challenges were encountered during the study, highlighting areas that require further development and consideration for industrial deployment.

- The inherent structural limitations of low-stiffness materials constrain the applicability of the gripper primarily to lightweight tasks such as plastering, painting, and general tool handling. Current grasping force and mechanical stability levels are insufficient for heavy load-bearing tasks, including the transportation of structural materials. To address these limitations, advanced hybrid designs that integrate rigid and soft components could be explored. Such designs would aim to enhance load-bearing capabilities without sacrificing the beneficial compliance characteristics of the existing soft gripper design.
- The durability of the soft hand gripper remains a critical limitation. Silicone elastomers, while advantageous for their compliance and safety properties in electrically hazardous or high-temperature environments, degrade through prolonged exposure to mechanical stress, chemical interactions, or environmental factors such as ultraviolet radiation. The resulting wear not only necessitates regular maintenance or component replacement but also impacts the reliability and predictability of the gripper’s performance over lifespan.
- A detailed cost–benefit analysis that includes material synthesis, scalability of manufacturing processes, and maintenance requirements is essential to assess the gripper’s industrial viability comprehensively. While silicone elastomers are expected to offer significant cost benefits, exact cost-effectiveness and long-term sustainability in high-volume manufacturing contexts remain to be demonstrated conclusively.

Regarding control and operation, the integration of a precise coordinate system remains a critical aspect. In the current implementation, the coordinate system can be engaged at the intersection point of the palm and fingers, functioning as the central reference point for grasp planning and execution. However, due to the elastic deformation and material degradation over time, there is a gradual shift or offset in this coordinate system. Continuous material wear results in changes to finger geometry, leading to positional inaccuracies during repetitive operations. Addressing this issue, it could involve periodic recalibration of the coordinate system or incorporating adaptive control algorithms capable of compensating for changes in the geometry resulting from wear. The construction industry’s comparatively unpredictable and dynamic nature poses unique challenges that differ significantly from other areas. However, referencing the automotive settings of the automotive industry highlights distinct potential insights. Automotive manufacturing environments have long embraced robotic automation, including the deployment of collaborative robots and precision-driven end-effectors suitable for repetitive tasks requiring high accuracy. Their collaborative solutions—such as sensor integration, adaptive control, and real-time feedback mechanisms—could be leveraged to enhance the functionality of soft robotic grippers in construction contexts. Cross-industry insights could significantly accelerate the development of practical and robust robotic systems tailored to the specific needs of construction environments.

## 4. Conclusions

This architectural robot study introduces an innovative biomimetic soft robot gripper designed for autonomous operation in architectural fabrication and building applications. The gripper’s unique features address several key challenges in the field, offering a cost-effective and efficient solution for robot-assisted construction tasks. The gripper functions without pneumatic power, sensors, or pre-programming, reducing complexity and operational costs. This design minimizes errors due to local collisions, contact with electrified objects, temperature fluctuations, and underwater operations. The gripper’s novel gripping mechanism incorporates a pre-stressed reversible gripping system that enables secure handling of multiple hand tools with a single end-effector. The maximized contact area enhances stability and reduces malfunctions caused by overinflation. Additionally, the integration of a synthetic SHM significantly enhances the gripper’s durability, mitigating damage from environmental factors and repetitive use, thus extending the gripper’s lifespan and reducing operational interruptions. While the soft hand gripper shows promise, several challenges remain. The gripper performs well with objects having a concentrated center of gravity but struggles with tilted or wider objects. Further research is needed to ensure stable operation under impact forces from tools like hammers and drills. Improvements in air pressure regulation, material durability, and precision control could expand the gripper’s capabilities for more complex tasks. The proposed biomimetic soft robot gripper represents a significant step forward in the development of versatile, resilient, and adaptable tools for architectural fabrication and building applications. While further refinements are necessary to address current limitations, the gripper’s innovative design and self-healing capabilities offer a promising foundation for future advancements in soft robotics for construction and beyond. This research not only contributes to the practical implementation of soft robotics in demanding environments but also paves the way for more efficient and safer human–robot collaboration in real-world construction settings.

## Figures and Tables

**Table 1:** Initial comparison of finger design feasibility.

	Type 1	Type 2	Type 3
Pneu-net Design (Section)	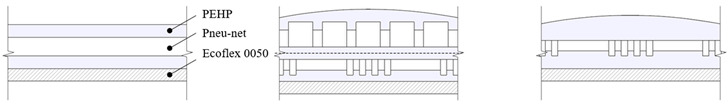		
Structure	Double layer Linear single chamber + PSRM elastomeric layer	Tri-layer 4 mm-width multi-chamber at the middle + 1 mm width multi-chamber + PSRM elastomeric layer	Double layer 1 mm-width multi-chambers + PSRM elastomeric layer
Test Configuration	Hook-shaped actuator with three and five branches in the opposite direction Simplest pneu-net design	Replicating the finger motion of bidirectional and sequential movement	Implementing an effective zero-power holding Fiber reinforcement for grasping rigidity
Limitation	Difficulty with hand-like design Homogeneity of chamber arrangement	Additional air injections are required for grasping Difficulty in layer attachment Slow shape recovery	

**Table 2:** Comparison of silicone finger gripper performance (non-prestressed, pneumatic).

**Design and Material**	**Source**	**Max. Tip Force**	**Max. Payload**	**Strain Capability**
Single Finger (L = 30 mm) KE-1606 RTV silicone rubber	[37]	0.17 N at 180 kPa	N/A	48° bending at 150 kPa
Multi-Finger (L > 200 mm) 3D-Printing polymer	[38]	N/A	5 kg at 300 kPa (four fingers)	>~100° bending at 200 kPa
Single Finger (L = 140 mm) Silicone rubber	[39]	N/A	0.6 kg at 0.1 MPa (single finger)	N/A
Single Finger (L = 119 mm) 3D Printing TPU	[40]	~0.5 N at 50 kPa 1.94 N at 150 kPa	Holds only light-weight objects	~25° bending at 30 kPa
Single Finger (L = 90 mm) Ecoflex™ 30	[41]	0.4~0.8 N (up) and ~1 N (down) at 30 kPa	N/A	175.2° bending at 40 kPa
Single Finger T00 + E600 Silicone	[42]	0.6 N at 50 kPa	~0.22 kg (four fingers)	90° bending at 21 kPa

## Data Availability

The data supporting the findings of this study are available upon request from the corresponding author and are also included in the Appendix A, Appendix B, Appendix C, Appendix D and Appendix E. In addition, the self-healing material property data presented in this study are openly available from Elsevier (Copyright 2023) at https://doi.org/10.1016/j.cej.2022.140543 (Accessed on 25 April 2025).

## Supplementary Materials

The following demonstration videos are available on the journal’s website and may be downloaded at: https://goo.su/GKsZMm (Video S1: 0_Self-healing-elastomer.mp4, Video S2: 1-1_Self-healing-capability.mp4, Video S3: 1-2_Finger_motion.mp4, Video S4: 2_Robot-plastering.mp4, Video S5: 3_Robot-paintbrush.mp4).

## Abbreviations

The following abbreviations are used in this manuscript:

PSRM	Pre-stressed reversible mechanism
SHM	Self-healing material
PEHP	Polyborosiloxane–Ecoflex hybrid polymer

## Appendix A

Procedure for 3D PEHP form fabrication: (1) synthesize the PBS solution. (2) Coat the 3D-printed mold with a PVP solution to prevent adhesion. (3) Prepare the Ecoflex™ polymer solution by mixing Ecoflex™ 30 Part A and Part B in a 1:1 weight ratio. Then, add the PBS solution to the Ecoflex™ solution at a 1:2 weight ratio and thoroughly blend the mixture using a mechanical stirrer for 5 min. (4) Immediately pour the blended liquid mixture into the PVP-coated 3D-printed mold. (5) Cure the mold in a vacuum oven at 60 °C for 24 h to complete the solidification.

## Appendix C

(1) Design the mold geometry using Rhino™. (2) Fabricate the mold via 3D printing using PLA filament. (3) Prepare two different types of silicone for respective mold sections and slowly pour them into the mold after degassing to remove air bubbles. (4) Cure the filled mold for 4 h, then demold the solidified elastomer. (5) Bond the segmented elastomer parts using liquid-state silicone and cure the assembly for 4 h additionally. (6) Prepare a prestretched elastomer sheet fixed onto the frame. (7) Attach the main body (fabricated in Step 5) onto the prestretched layer using the same silicone. After a further 4-h curing, cut along the outer contour. (8) Insert the fingers into the joint cavities and connect them with urethane tubing. Assemble all components to complete the soft hand gripper.
